# Vapor–solid–solid growth dynamics in GaAs nanowires†

**DOI:** 10.1039/d1na00345c

**Published:** 2021-08-05

**Authors:** Carina B. Maliakkal, Marcus Tornberg, Daniel Jacobsson, Sebastian Lehmann, Kimberly A. Dick

**Affiliations:** Centre for Analysis and Synthesis, Lund University Box 124 22100 Lund Sweden carina_babu.maliakkal@chem.lu.se; Solid State Physics, Lund University Box 118 22100 Lund Sweden; NanoLund, Lund University Box 118 22100 Lund Sweden; National Center for High Resolution Electron Microscopy, Lund University Box 124 22100 Lund Sweden

## Abstract

Semiconductor nanowires are promising material systems for coming-of-age nanotechnology. The usage of the vapor–solid–solid (VSS) route, where the catalyst used for promoting axial growth of nanowires is a solid, offers certain advantages compared to the common vapor–liquid–solid (VLS) route (using a liquid catalyst). The VSS growth of group-IV elemental nanowires has been investigated by other groups *in situ* during growth in a transmission electron microscope (TEM). Though it is known that compound nanowire growth has different dynamics compared to elemental semiconductors, the layer growth dynamics of VSS growth of compound nanowires have not been studied yet. Here we investigate for the first time controlled VSS growth of compound nanowires by *in situ* microscopy, using Au-seeded GaAs as a model system. The ledge-flow growth kinetics and dynamics at the wire–catalyst interface are studied and compared for liquid and solid catalysts under similar growth conditions. Here the temperature and thermal history of the system are manipulated to control the catalyst phase. In the first experiment discussed here we reduce the growth temperature in steps to solidify the initially liquid catalyst, and compare the dynamics between VLS and VSS growth observed at slightly different temperatures. In the second experiment we exploit thermal hysteresis of the system to obtain both VLS and VSS at the same temperature. The VSS growth rate is comparable or slightly slower than the VLS growth rate. Unlike in the VLS case, during VSS growth we frequently observe that a new layer starts before the previous layer is completely grown, *i.e.*, ‘multilayer growth’. Understanding the VSS growth mode enables better control of nanowire properties by widening the range of usable nanowire growth parameters.

## Abstract 2

The dynamics of layer growth in nanowires with a solid catalyst is compared to that with a liquid catalyst.

## Graphical abstract

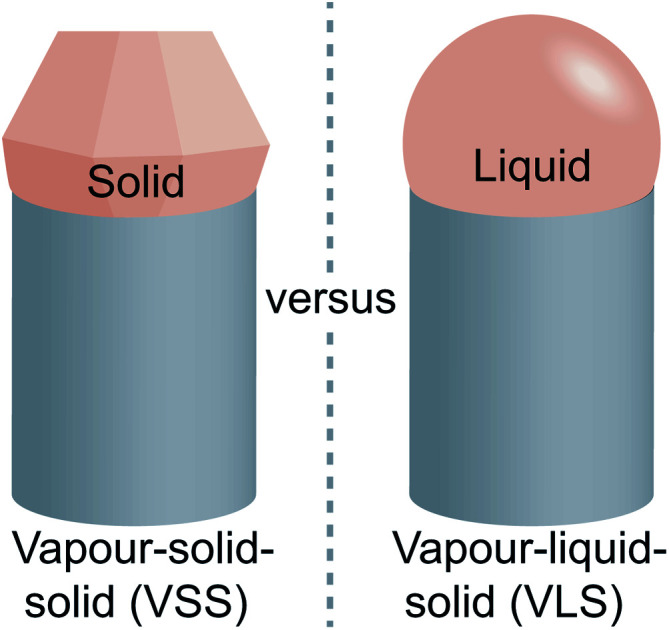

## 1. Introduction

Controlling the electronic, mechanical and optical properties of semiconductor nanowires by tuning their crystal structure, composition and morphology enable their application in, for instance, nano-electronic, optoelectronic and energy harvesting devices.^1–4^ Precise understanding and control of the dynamics of the crystal growth process are in turn key to tuning these important parameters. Nanowires are most often grown using a foreign liquid metal to promote the anisotropic one-dimensional growth. This process occurs by the ‘vapor–liquid–solid’ (VLS) mechanism;^5,6^ accordingly, the atomic species constituting the semiconductor dissolve in the seed particle, form a supersaturated metallic liquid alloy (‘catalyst’), and subsequently precipitate to form the solid semiconductor.^5^ An alternative vapor–solid–solid mechanism (VSS), in which the catalyst is a solid instead of a liquid, has also been proposed.^7,8^ Some nanowire growths have been identified to occur with a solid catalyst on the basis of *ex situ* characterization of catalyst post-growth;^9–12^ or by investigating the temperature range required to grow the wires^13^ combined with equilibrium phase diagrams. However, effects such as size dependent decrease in melting point,^14–17^ super-cooling^18–21^ and thermal history^18^ pose challenges to an accurate assessment of the catalyst phase in *ex situ* studies. Later on, *in situ* transmission electron microscopy (TEM)^18,22–25^ and *in situ* reflection high-energy electron diffraction (RHEED)^26^ studies have provided direct observation of VSS growth. It is thus very important to understand how the VSS process works and how it compares to the well-studied VLS growth process.

One major advantage of VSS growth compared to VLS is that in some material systems it can enable fast switching of materials in axial nanowire heterostructures.^22^ During VLS growth the liquid catalyst acts as a reservoir of the nanowire species, and hence the switch from one composition to another is gradual.^27–29^ On the other hand, the solubility of nanowire species in the solid catalyst particle is lower, enabling abrupt junctions, increased purity and better control.^22,26,30^ Another advantage of VSS growth is that it greatly expands the range of possible catalyst materials, to include those with inappropriately high eutectic temperatures but potential advantages to, for instance, crystal structure control and incorporation of trace elements.^31^ This would also enable the fabrication of nanowire-based devices in a way that is compatible with standard industrial processes.^32–34^ Another interesting realm could potentially be where the catalyst can simultaneously have solid and liquid parts coexisting; this could for example enable a direct growth of embedded compositional quantum dots within a wire due to the difference of solubility in the two phases.

It is understood that nanowires mostly grow layer-by-layer along the nanowire–catalyst interface (for both VSS and VLS growths).^35,36^ The growth of each ledge, consisting of either one or more atomic layers, is often referred to as ‘ledge-flow’ (and sometimes also as ‘step-flow’). A few groups have investigated the VSS growth of nanowires *in situ* in a transmission electron microscope (TEM),^18,22–25,37,38^ a couple of which had a spatial resolution to directly measure the height of individual ledges.^23,38^ Hofmann *et al.* observed during *in situ* VSS growth of Si nanowires that new ledge(s) can form even before the first ledge is completed, and that each ledge can be made of more than one atomic layer.^23^ On the other hand, VSS Si-nanowire studies from another group reported that each ledge was only one bilayer thick (found indirectly due to limited spatial resolution).^24,25^ They occasionally observed steps of triple bi-layer height when the surface was unclean or for a small diameter nanowire that grew slowly even at higher precursor partial pressure.^38^ On comparing VLS (Au-catalyzed) and VSS (Cu_3_Si-catalyzed) Si nanowire growth happening at roughly the same growth rates (but different growth conditions), they observed that during VLS growth each bilayer grew rapidly once nucleated, but with long waiting time in between successive layer growths. On the contrary, in the VSS case the individual bilayers grew slowly but with short waiting times between successive layers.^24^

Note that the above-mentioned studies on layer growth dynamics in VSS systems were on elemental semiconductors – either Si or Ge nanowires.^22–25^ Although Si and Ge are very important semiconductor material systems (especially for electronics), as they are indirect bandgap materials they are inappropriate or less than ideal for certain optical applications. Many compound semiconductors, on the other hand, have a direct bandgap making them more efficient in such applications.^39^ Hence it is very important to understand growth of compound nanowires. However, the layer growth dynamics in compound nanowires are different from those in monoatomic nanowires. For the VLS growth of a binary nanowire using a (foreign) metal seed, the two different component species could have very different miscibilities in the metal catalyst, in turn affecting the kinetic processes.^40^ Naturally this implies that the VSS growth of compound nanowires could be different from VSS growth of monoatomic nanowires. Therefore, it is important to separately study VSS growth of compound nanowires and compare the dynamics to the VLS route. However, such an investigation has not been reported yet.

We report here for the first time direct *in situ* observation of the layer growth dynamics during VSS growth of a compound nanowire. The aim of this study is to compare the growth dynamics of the VLS and VSS processes using the same catalyst material and similar growth conditions. Growth is performed in an aberration-corrected environmental TEM and observed *in situ* and *in operando*. We use two strategies in this study: (a) reducing growth temperature in steps and (b) cool the system followed by heating it to obtain solid and liquid catalysts at the same temperature, while in both cases keeping the precursor flow and the starting seed metal (Au) the same. The layer growth data studied for different temperatures for the first strategy is from one individual GaAs nanowire. The second strategy is employed using another single nanowire. We observe that the growth dynamics of GaAs are significantly different from the case of monoatomic VSS nanowire growth. We also find that the growth rate during VSS growth need not necessarily be significantly different from VLS growth under similar conditions.

## 2. Results and discussion

### 2.1. Nanowire growth

GaAs nanowires were grown *in situ* in an environmental TEM. TEM chips based on silicon nitride (amorphous) act as a substrate and heater. The chips are engineered to have regions where the SiN_*x*_ is etched out for improved spatial resolution. Au nanoparticles were deposited on the chips to seed the growth. The chips were heated to 420 °C and the precursor gases (trimethylgallium for Ga; arsine for As) were introduced to nucleate and grow the nanowires using MOCVD (metal–organic chemical vapor deposition). At 420 °C these nanowires grow by the VLS mechanism. Although we observed VSS growth for many nanowires, we have chosen to focus in this article on two examples where it was possible to vary the growth parameters widely yet with small temperature steps to extract meaningful quantitative data and make relevant comparisons, while simultaneously avoiding the effects of inter-wire variations. In the first experiment discussed here the temperature was decreased in steps to solidify the catalyst and the layer growth was studied at different temperatures – *spanning VSS and VLS growth. The second is an experiment where we initially record VLS growth at a particular temperature, then decrease the temperature drastically to solidify the catalyst and then return to the earlier studied temperature to compare VLS and VSS growths at the same temperature.

### 2.2. Stepwise cooling

**Fig. 1:**
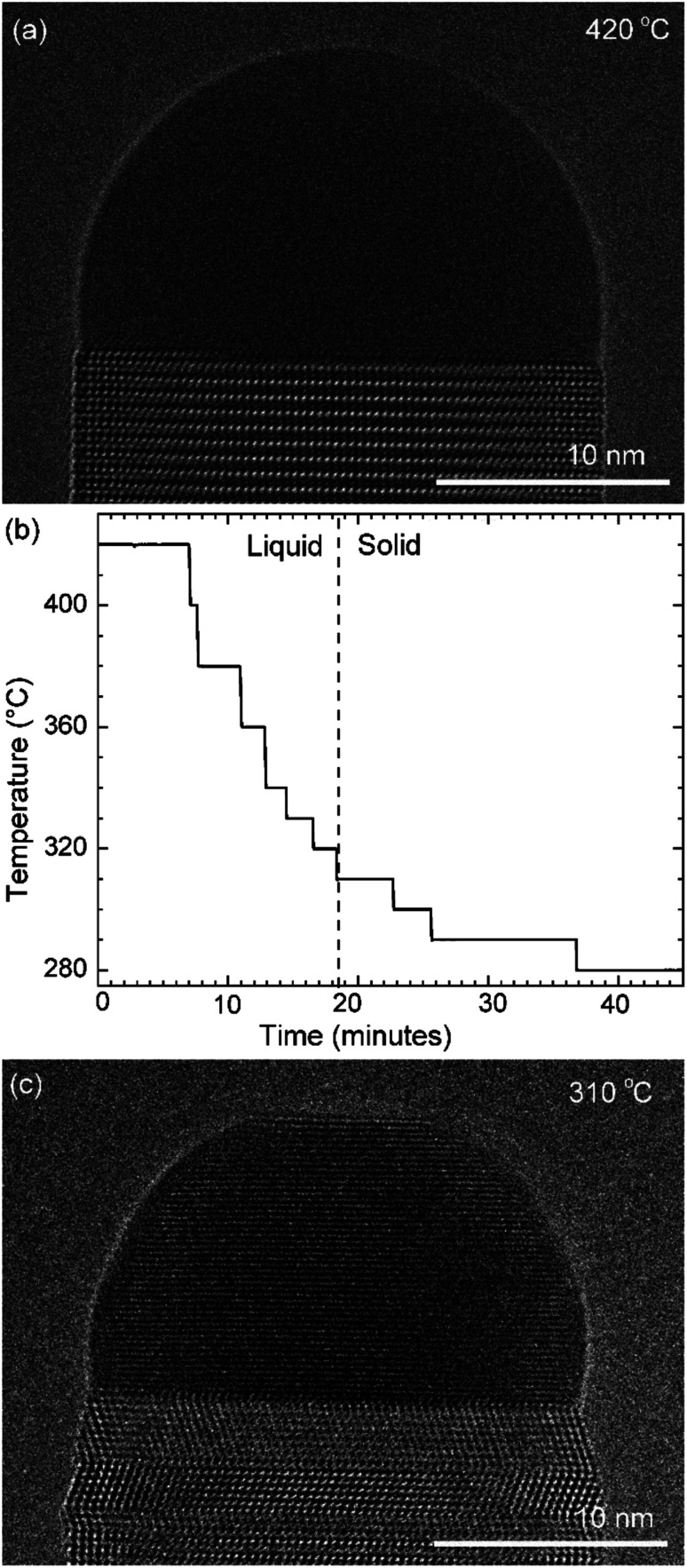
Stepwise cooling: (a) HR-TEM image of the nanowire–catalyst system during layer growth at 420 °C. At this temperature the catalyst is liquid. (b) The growth temperature was decreased in steps in this experiment. The catalyst solidified at 310 °C with one set of the crystal planes aligned parallel to the nanowire–catalyst interface. (c) TEM image where the catalyst has solidified.

First let us discuss the experiment where the temperature was decreased in steps. The nanowire was nucleated at 420 °C and was grown by the VLS mechanism (Fig. 1a) with a diameter of 22 nm. The growth temperature was then decreased in steps (Fig. 1b), and at each temperature videos of the nanowire growth were recorded. At 310 °C the particle solidified (Fig. 1c) while the growth continued (solidification was identified by the observation of a lattice structure in the catalyst). The observed lattice spacing in the catalyst particle (0.25 nm) is consistent with the Au–Ga α′ phase (∼13% Ga). At temperatures between 420 °C and 330 °C the nanowire grew in the wurtzite structure and the catalyst–nanowire interface diameter did not change noticeably. At 320 °C and lower the wire grew in the zinc blende structure. Note there was no change in the crystal structure observed between VLS and VSS growth at similar temperatures (320 °C VLS and 310 °C VSS), and so this change to zinc blende at 320 °C is a consequence of temperature rather than the catalyst phase.

**Fig. 2:**
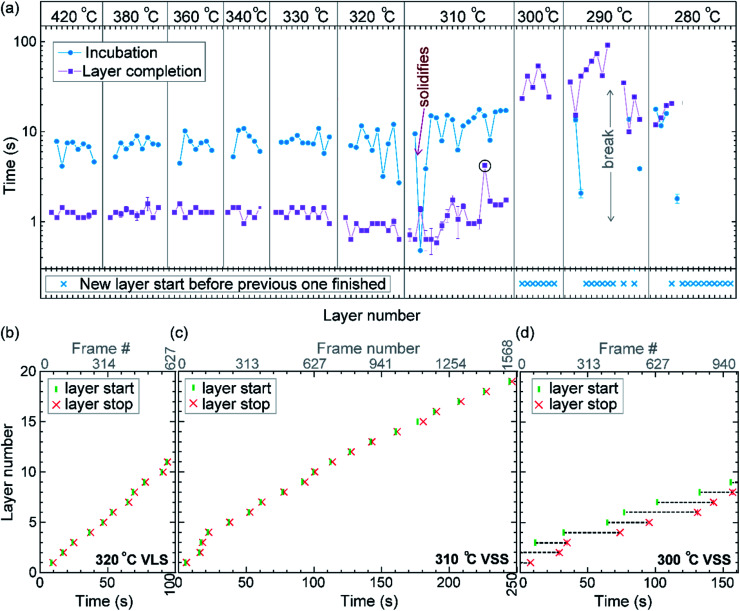
Layer growth at different temperatures with solid and liquid catalysts: (a) incubation time and layer completion time during growth at different temperatures in the top panel. The *x*-axis is the layer number plotted forward in time, but is discontinuous when the temperature is decreased. Each *x*-axis tick indicates one layer. Temperature is noted at the top. When a new layer starts before the previous layer finishes it is marked with a cross in the bottom panel. The starting and ending of each layer for successive layers are plotted for (b) VLS growth at 320 °C, (c) VSS growth at 310 °C, and (d) VSS growth at 300 °C. The *x*-axis shows the time from the start of each video. During 300 °C VSS growth new layers were seen to nucleate even before the previous layer is completely grown. In (b)–(d) the *y*-axis range is set to be the same.

We call the time each layer takes to complete since its nucleation as the ‘layer completion’ time (also sometimes called step-flow time in the literature), and the difference between the ending of one layer and the start of the next layer as ‘incubation time’. The incubation time and layer completion time were measured from the videos at each temperature and are plotted in Fig. 2a, incubation time is shown as blue circles and layer completion time as purple squares. The *x*-axis of the plot is the layer number in ascending order but is discontinuous wherever the temperature is decreased. The incubation time before nucleation of each layer is plotted at the same *x*-value where its layer completion is positioned in Fig. 2a. Sometimes a new layer starts to grow even before the previous layer has formed fully. When this occurs, it is denoted by a cross mark in the bottom panel of Fig. 2a; this representation however does not give information about how far into the growth of the previous layer the new layer started. The information on how early or late during the growth of the previous layer(s) that the new layer starts can be visualised using Fig. 2b–d (in addition to data in Fig. 2a, plots b–d also contain information of additional layers for which only part of the layer growth process was recorded, *i.e.* those at either the beginning or the end of each video). The time we started recording videos at each temperature is set to 0 s for that temperature in Fig. 2b–d.

In the VLS mode the layers grew one at a time *i.e.* a new layer starts to grow only after the previous one has completely grown (Fig. 2a and b); there were no occasions of double or multilayer growth. The time it takes for each individual layer to grow (layer completion) was very similar for 420, 380, 360, 340 and 330 °C where the catalyst was liquid, about 1.3 s. As the temperature is decreased from 420 to 320 °C, there is a very gradual increase of the incubation time (averaged value is plotted in ESI Fig. S1†). At 320 °C, where the crystal structure switches to zinc blende, the layer completion becomes faster (a transition to zinc blende structure at lower temperature is consistent with previous reports^41^). On further decreasing the temperature to 310 °C the first layer grew while the catalyst was still a liquid. At the start of the next layer (in the same video frame) the catalyst solidified with one set of lattice planes aligned parallel to the nanowire catalyst interface. The first two layers that nucleated after the catalyst solidified had a shorter incubation time compared to the later layers (Fig. 2a and ESI Fig. S2†). This can be attributed to a transient effect, as the particle initially has a higher supersaturation due to excess Ga just after solidification (according to the phase diagram^42^ Ga solubility in the solid phase is lower than the Ga concentration of liquid Au–Ga). Since the layer completion is expected to be limited by As availability^40^ (due to the low solubility of As in the Au–Ga system^36,42–46^), the excess Ga is not expected to influence the layer completion time.

The layer completion times for the solid catalyst at 310 °C, and liquid catalyst at 320 °C are similar, implying that the diffusion through the catalyst is not the rate limiting step here (because diffusion through the solid phase is expected to be orders of magnitude slower than that through a liquid catalyst).^9^ At 310 °C most of the growth occurred as single layers (each layer finished growing before the next one started). However, there was one instance of a double layer growth (indicated by the black ellipse in Fig. 2a) that happened when the new layer(s) was twinned relative to the crystal orientation below (Fig. S2†). This layer grew extremely slowly, suggesting that in case more than one layer is growing in parallel, then each of those layers would grow slow. This is reasonable as the layer growth rate is proportional to the rate of arriving As atoms^40^ and the time to complete each layer(s) naturally depends on the number of atoms required to form the layer(s). On decreasing the temperature further to 300, 290 and 280 °C, the catalyst remained in the solid phase. At these temperatures there were several instances of multiple layers growing simultaneously and each layer grew very slowly. The transition from a (mostly) single layer growth at 310 °C to a regime where more than one layer can grow simultaneously by a difference of just 10 °C indicates that the growth is very sensitive to different growth parameters. At 280 °C, multiple layers nucleated before the previous finished, followed by an evident displacement of the catalyst to the right side. Since this displacement interferes with accurate interpretation of the image data, the experiment was terminated at this point; only data for layers that started before this displacement are included in the plot (Fig. 2a), and so completion time for these layers is not measured.

**Fig. 3:**
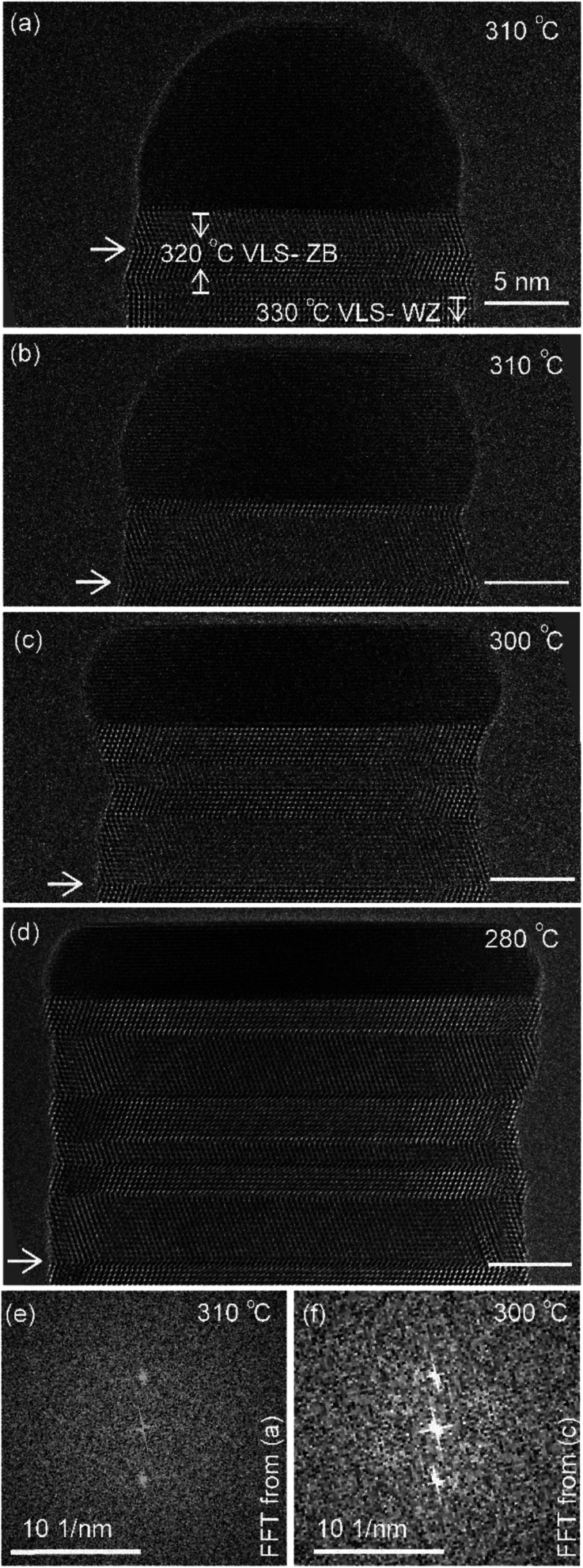
(a–d) TEM images (after averaging and rotating) of the nanowire at the same magnification at different temperatures. The arrow marks the same defects in all the images. Scale bar is 5 nm in (a–d). Power spectrum/Fast-Fourier-Transform (FFT) of the catalyst region from images (a) and (c) are given as (e) and (f) respectively.

The diameter of the catalyst–nanowire interface did not change significantly while the wire was growing in the wurtzite structure (400–330 °C, diameter was ∼22 nm). However, when this nanowire was growing in the zinc blende structure, a change in the diameter of the nanowire–catalyst interface was observed as the growth progressed. During the VLS zinc blende growth (at 320 °C), the interface diameter decreased (from 21.8 nm to 21.2 nm). But in the VSS mode (zinc blende structure) the diameter kept increasing gradually and the catalyst height was subsequently decreasing at all investigated temperatures. Fig. 3 shows a few representative TEM images. Quantitative data on the change of interface diameter as a function of time are shown in ESI Fig. S4.† There was no apparent change in the lattice structure of the catalyst during VSS growth (refer Fig. 3e and f). This reshaping thus changes the relevant dimensions of the system including the GaAs–catalyst interface area, exposed surface area of the catalyst, the nanowire sidewall surface area and perhaps the catalyst volume – which in turn affects the amount of Ga and As required to form each layer, catalytic decomposition of the precursor species at the catalyst surface, the diffusion of the growth species in the catalyst (be it through the catalyst volume or through the catalyst–wire interface), collection of Ga adatoms from the sidewalls of the nanowire, *etc.*

**Fig. 4:**
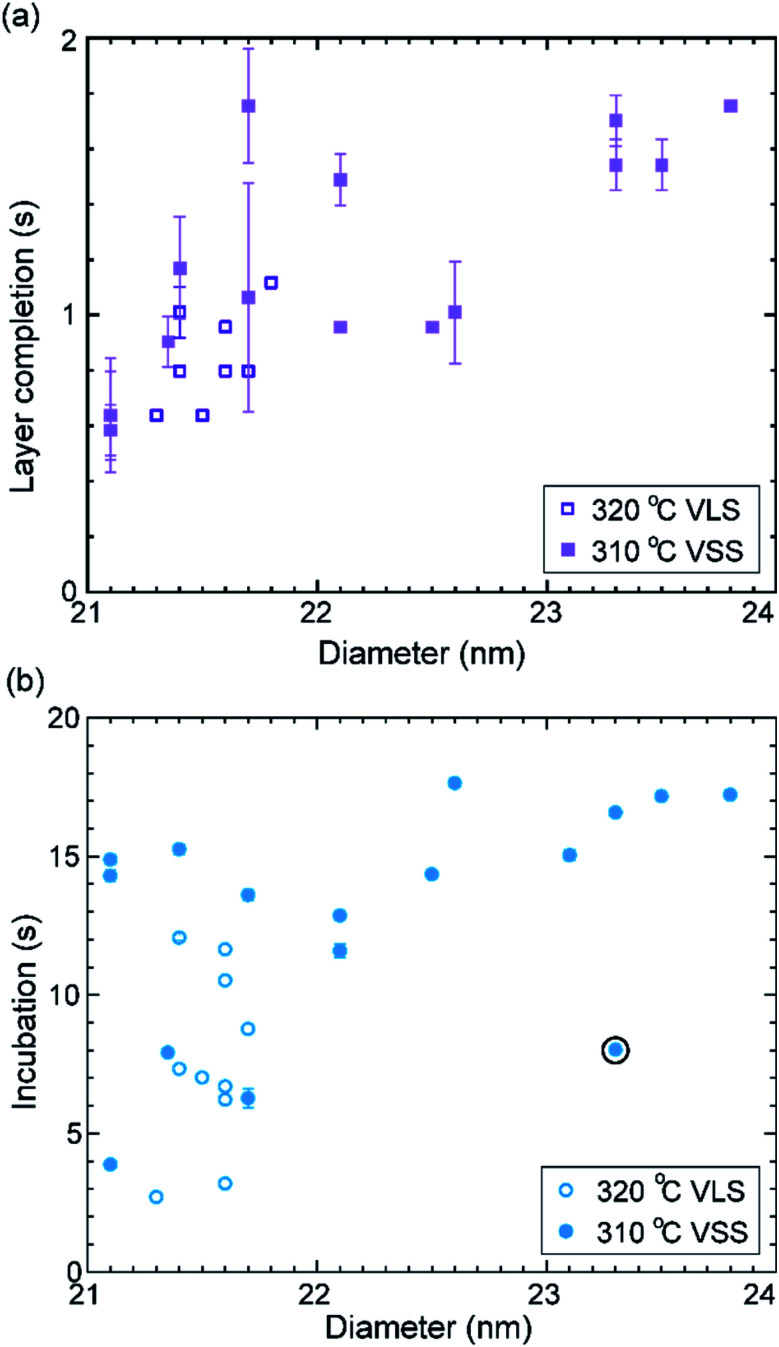
Growth times and diameter: the layer completion time (a) and incubation time (b) are plotted against the nanowire diameter measured at the catalyst–nanowire interface. Data from two nearby temperatures where the catalyst phase changed are used here. Solid symbols denote layer growth times for the solid catalyst particle at 310 °C and the open symbols denote the liquid catalyst at 320 °C. The range in plot (a) does not include the layer completion time of the twinned double layer (value 4.25 ± 0.1 s) that grew. The incubation time right after this double layer was lower and is encircled in the plot (b). The error bars are related to the uncertainty in identifying the starting and stopping of each layer from the video.

Let us take a closer look at the layer growth times at the two temperatures across which the solidification occurred (*i.e.* 320 and 310 °C) in association with the diameter change. Fig. 4 shows layer completion time and incubation time as a function of the diameter of the wire–catalyst interface (*y*-axes are in the linear scale) (the first couple of layers which showed a transient behaviour just after solidification at 310 °C are excluded from this plot). We see that for a similar diameter both the layer completion time and the incubation time are similar for the 320 °C VLS and 310 °C VSS growths. As discussed earlier, the diffusion through solid phases is typically orders of magnitude lower than through a liquid. Our observation of similar layer completion times for the solid and liquid catalyst phases implies that the diffusion to the growth front, irrespective of whatever the diffusion route can be, is not the rate limiting step.^9^ With increasing diameter, we see that layer completion becomes slower. The layer completion is limited by the availability of As atoms in/at the catalyst.^40,47^ Arsenic atoms reach the catalyst by direct impingement on the catalyst surface the (rather than primarily by surface diffusion of As adatoms along the nanowire side facets to the catalyst).^48^ One possible explanation for the increase of layer completion time with increasing diameter could be related to the increase of ratio between the interface area to the exposed catalyst surface area (more details of this model considering geometric parameters are in ESI Section S4†). Moreover, the nanowire width increases significantly under these conditions indicating that there could be competition for the available growth material between the axial and radial growths. This will increase the incubation time, and perhaps increase the layer completion time.

**Fig. 5:**
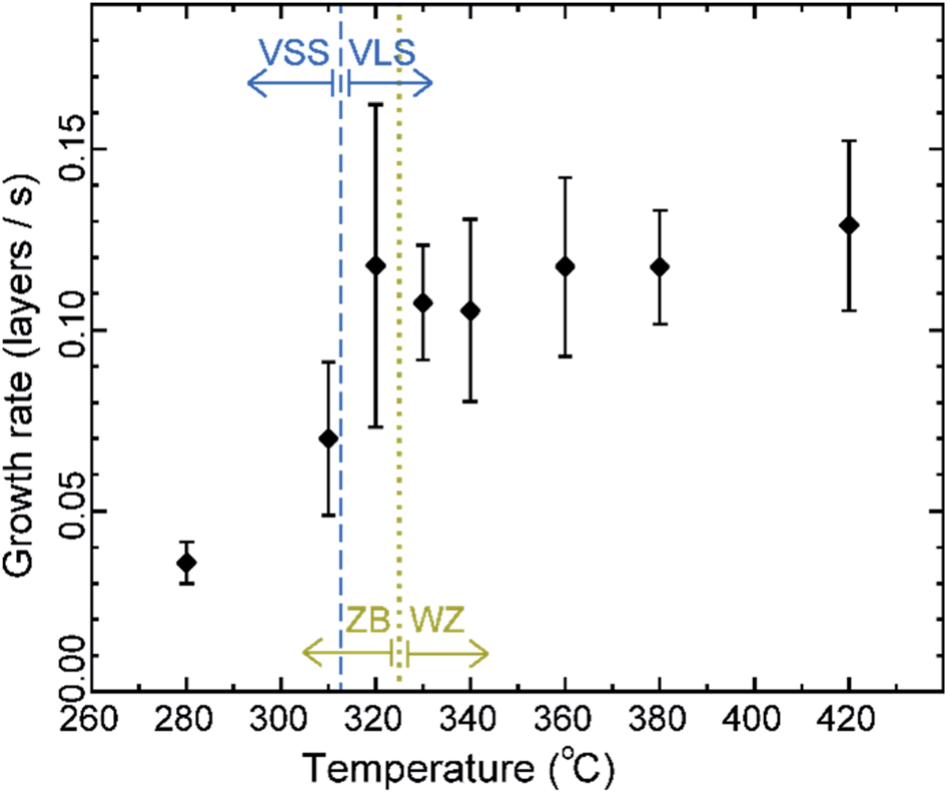
Average growth rate: average growth rate of the same nanowire at different temperatures. At 310 °C and lower the catalyst is solid. The wire was growing in the wurtzite (WZ) structure at temperatures between 420 °C and 330 °C and as zinc blende (ZB) at lower temperatures. Error bars represent standard deviation.

The average growth rates at the different temperatures studied are plotted in Fig. 5 (details of the measurement are given in the Methods section). We can see that in the VLS mode there is a small decrease of growth rate with decreasing temperature, but there seems to be a relatively larger decrease of average growth rate at 310 °C when the growth mode switched to VSS (the comparison here is between (a) the observed VSS growth at 310 °C and (b) a linearly extrapolated value for hypothetical VLS growth at 310 °C based on the VLS growth at higher temperatures). Some earlier reports studied monoatomic nanowire growth and reported one or two orders of magnitude slower growth rate for VSS compared to VLS.^18^ However, in this study of GaAs compound nanowires the observed difference between VLS and VSS nanowire growth rate is not as drastic. Moreover, note that the catalyst reshaping and nanowire widening will also influence the average growth rate. As discussed earlier, the layer completion time and the incubation time for the 320 °C VLS and 310 °C VSS growths are very similar (Fig. 4) for a similar nanowire diameter. This in turn implies that the growth rates (which is essentially inverse of the sum of incubation and layer completion times) are also very comparable between 320 °C VLS and 310 °C VSS for comparable dimensions. Thus, the difference between the average growth rate in the VLS and VSS case observed here is at least partly due to the change in morphology.

In this experiment the VLS and VSS growths are compared at different temperatures. Temperature can change different parameters like (gas phase) thermal decomposition of precursors, Ga adatom diffusion on NW sidewalls, Ga and As solubility in the catalyst, As evaporation rate, flow patterns in the growth cell and surface energies. It is also possible to compare the VLS and VSS at the same temperature by exploiting thermal hysteresis. This approach also has a limitation—that it relies on a transient state and not a steady state phenomenon. Hence it is important to use both these approaches and compare the results. The second approach of thermal hysteresis is what we discuss next.

### 2.3. Experiment utilizing thermal hysteresis

**Fig. 6:**
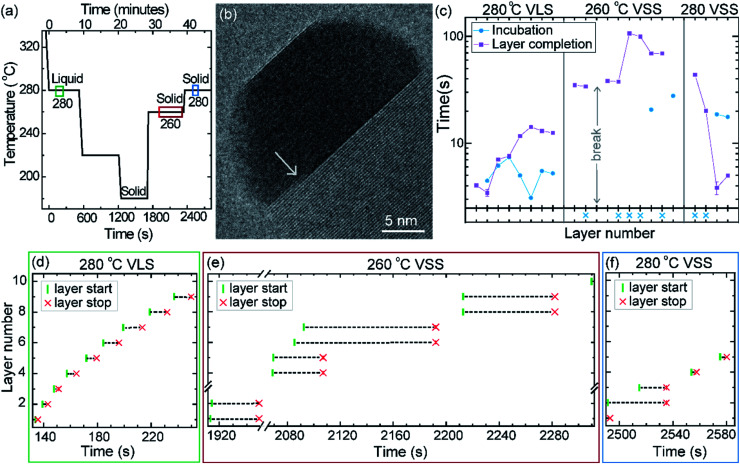
Thermal hysteresis experiment: (a) sample temperature as a function of time. The three colored boxes correspond to the time interval used for data in sections (d)–(f). (b) TEM image of the nanowire–catalyst system during VSS growth at 280 °C. In this TEM micrograph we see two layers growing simultaneously (marked by the arrow). (c) Incubation time and layer completion time for each layer during VLS growth at 280 °C and VSS growth at both 260 °C and 280 °C. The *x*-axis is discontinuous at the temperature changes. Each tick on the *x*-axis indicates one layer. The starting time (green vertical line) and ending time (red cross) of each layer is denoted for successive layers during the (d) VLS growth at 280 °C, (e) VSS growth at 260 °C and (f) VSS growth at 280 °C. We see in the VSS cases that often a new layer nucleates while the previous layer is still growing. In (e) we can also see occasions where a double layer has nucleated simultaneously. In (b)–(d) the *x*- and *y*-axis scales are set to be the same across panels.

The phase of the catalyst is a function of not just the temperature but also the thermal history or hysteresis.^18^ In the second experiment we use this to obtain VSS and VLS growth at the same temperature. Nucleation of the nanowires was performed at 420 °C, just like the previously discussed experiment. The diameter of this nanowire was 27 nm. After nucleation the growth temperature was decreased directly to 280 °C without pausing at intermediate temperatures to allow the system to reach a steady state (Fig. 6a); this procedure enabled the catalyst to remain a supercooled liquid at this lower temperature. We set time = 0 when the sample temperature reached 280 °C (on decreasing from 420 °C) and use this time reference in Fig. 6. After observing some VLS layer growth events at 280 °C the temperature was further decreased in steps as shown in Fig. 6a, to hasten solidification of the particle. From this low temperature where the particle was a solid, the temperature was increased to 260 °C and then again to 280 °C and the VSS growth was monitored. Representative segments of the video recording showing VLS and VSS growths at 280 °C are given as ESI Videos,† after some processing. A few more representative images are shown in ESI Section S5.† The observed lattice spacing (0.22 nm) in the solid catalyst is again consistent with the Au–Ga α′ phase (∼13% Ga), similar to the earlier discussed stepwise cooling series. In this experiment the diameter changes were rather small (± 1–2 nm) and we did not see any significant catalyst reshaping as in the first experiment. In that stepwise cooling series with a smaller Au seed size perhaps there was some effect of the high surface-to-volume ratio on the energy balance. Other probable explanations for not seeing catalyst reshaping in the hysteresis series could be related to earlier formation of favorable facets or that the rearrangement of the solid was hindered by the lower temperature at which solidification occurred.

The layer completion times and incubation times for VLS growth at 280 °C, VSS growth at 260 °C and VSS growth at 280 °C are shown in Fig. 6c (as for the previous experiment, when a new layer starts to grow even before the previous layer has formed fully the incubation time is denoted by a blue crossing Fig. 6c). During VSS growth at 260 °C there are instances where double layers have nucleated simultaneously. The occurrence of double layers is not characteristic of specific temperature, but it has a certain probability that is related to temperature along with other parameters. The layer completion times at 280 °C VLS and VSS are comparable for single layers, although double layer growth occurs more slowly as previously observed. The incubation time during VSS growth is higher than that during VLS growth here at 280 °C. At a slightly lower temperature (260 °C) while the catalyst was a solid there were several instances where more than one layer was growing. We can also see an instance where 4 layers were growing in parallel (Fig. 6e).

## 3. Discussion

Some key features observed in the above experiments are discussed here. However, these discussions and conclusions are supported by observations from other VSS nanowire growths studied during the course of this study.

### 3.1. Comparison with monoatomic nanowire studies

In monoatomic VLS systems the layer completion was observed to be effectively instantaneous while the incubation was very long.^22,24^ The material required to supersaturate the system was sufficient to form one complete layer instantly once nucleated in that case. On the contrary, in monoatomic VSS cases the incubation time became very short and the layer completion time became long.^22,24^ The solubility of the nanowire species in the solid catalyst particle was low and thus a small excess was enough to supersaturate the catalyst and start a new layer^22,24^ (this low solubility in the solid catalyst is often the case, though not true universally for all systems). Such a drastic contrast of incubation and layer completion times between VLS and VSS reported in monoatomic nanowires is not observed in this study of compound (GaAs) nanowires.

Even for the VLS route, the layer growth dynamics in compound nanowires are significantly different from monoatomic nanowires.^40^ In the VLS growth of binary nanowires (GaAs studied as an example), the two elements forming the nanowire dissolve in the metal catalyst very differently.^42^ Indeed it is common for binary semiconductors that one of the constituents alloys more easily with the seed catalyst, while the other constituent could have a much lower solubility. Specifically, it is common that the metallic element (group II and III elements such as Ga) alloys readily with frequently used seed metal (*e.g.* Au), while most group V or VI elements, such as As, has poor miscibility in the catalyst.^49^ Under typical growth conditions this implies that the nucleation of a new layer is triggered by the chemical potential of the more miscible species, but the layer completion of each layer is restricted by less miscible species due to the scarcity of available atoms.^49^ Layer completion in compound nanowires even for VLS growth is not instantaneous;^40^ unlike the instantaneous layer completion in the VLS monoatomic nanowire growth case.^24^ It is thus not surprising to see even qualitative differences between compound and monoatomic nanowires for the VSS growth mode as well. Unlike seen in earlier reports on monoatomic NWs,^22,24^ the VSS incubation time observed here for GaAs in the single layer growth regime is not necessarily shorter than the VLS case, especially when accounting for changes in the nanowire diameter. Incubation time on the other hand is typically a bit longer for VSS GaAs than for VLS (in cases where layers grow one at a time).

### 3.2. Growth rate difference between VLS and VSS

Let us next discuss about the overall growth rate of VSS growth of nanowires in comparison to the VLS growth. Often in the literature VSS growths are assumed to be slower than VLS growth, sometimes by one or two orders of magnitude. In their report on *in situ* Au-catalyzed Ge nanowire growth Kodambaka *et al.* mentioned that the VSS growth rate was one or two orders of magnitude slower (when temperature and precursor pressure were kept the same, while the thermal hysteresis was exploited to obtain VLS and VSS data at the same temperature).^18^ However there are also reports with a much smaller difference. Chou *et al.* reported VSS growth of Si using Au–Ag catalysts to be a couple of times slower than VLS growth (when the experiment was also done exploiting thermal history).^30^ Comparable growth rates for VLS and VSS have been reported on the basis of *ex situ* growth studies also.^50,51^ In our study of GaAs nanowire growth, the VSS growth seems to be slightly slower than VLS growth for comparable growth conditions. How different the VSS growth rate is in comparison to VLS however depends on the specific chemical system, growth parameters and limiting processes (including precursor decomposition, diffusion *etc.*). Sometimes people assume VSS growth to be an impractically slower and thus inefficient process, and the idea of growing *via* VSS is often discarded even for systems that might benefit from the use of VSS over VLS. This is partly because of inappropriate comparisons in the literature among very different material systems or drastically different growth conditions. This current study on a single material system with small temperature steps to make relevant comparisons demonstrates the assumption of slow VSS growth to be not universally true. We believe that this study will encourage others to consider VSS growth as a means to obtain controlled growth of semiconductor nanostructures.

### 3.3. Multiple layers growing simultaneously in GaAs VSS growth

*In situ* TEM investigations reported on VLS growth observed layers growing only one after the other^22,24,52^ (except in one case of GaN nanowire growth^53^ which will be briefly addressed below). On the other hand, for the VSS growth of monoatomic nanowires, multiple layers growing simultaneously have been observed in the case of Si nanowires^23–25^ and the growth was predicted to happen at low ‘step mobility’.^24^ With the terminology used in this report this means a long layer completion time. In the GaAs experiments studied here we never observed more than one layer growing at any time during VLS growth. But in the VSS case we frequently observe that a new layer starts to grow even before the earlier layer finished forming. We also observe that in the VSS case nucleation of a double layer is also possible (*i.e.* we see two layers starting ‘simultaneously’ within the video resolution). Gamalski *et al.* showed that double layers could grow together if the line energy of a ledge with two layers is appropriately low.^53^ We also observe occasions where in spite of long layer completion times a new layer has not started (see the first few layers at 280 °C in Fig. 2a). This could be either due to nucleation being a stochastic (or probabilistic) phenomenon, or that the slow step mobility cannot be the sole reason for multilayer growth. To explain how multiple layers can grow in parallel we propose a heuristic model based on energetics, material availability, and most importantly different solubilities in the liquid *versus* solid catalyst.

In the VLS case, nucleation of each layer occurs only after overcoming a significant nucleation barrier; since the total number of atoms in the catalyst is small, the formation of a nucleus reduces the supersaturation sufficiently to prevent the formation of a new nucleation before the layer has finished growing. In the case of a solid catalyst, the solubilities of both Ga and As in the catalyst are very low. Thus, any extra Ga or As increases chemical potential significantly, seemingly much higher than the nucleation barrier. In such a scenario, the nucleation barrier becomes effectively less important and the requirement of a certain ‘critical’ supersaturation is not a major bottleneck. The rate limiting step is thus dominated by the sticking coefficient (or attachment rate) and material availability. This enables the starting of a new layer at the periphery due to the material supply directly from the vapor and additionally because it acts similar to a ‘defect’ or ‘kink’ site. The attachment process is still more favorable at the already growing layer than as a fresh layer at the rim of the catalyst–nanowire interface (we know this since we do not see several multilayers only just starting at the sides without growing into full layers). However, as the nucleation barrier becomes negligible for VSS, attachment of just a few atoms at the periphery can be sufficient to start a new layer, and there is a finite probability of this occurring before the already-growing layer is complete. This makes multilayer growth relatively more probable in VSS than in VLS. A possible explanation for the VSS growth at 310 °C showing a layer after layer growth behaviour could be the higher temperature compared to the other VSS temperatures studied here – as the temperature increases the effect of each individual Ga atom on the chemical potential decreases, increasing the nucleation barrier and the potential for the growth to occur in a purely nucleation-limited regime. We would like to emphasize that the multi-layer growth is not a fundamental feature of VSS growth, rather it becomes more probable during VSS due to the much-decreased solubilities of the semiconductor components. Among all the reports of VLS growth of different systems studied by *in situ* TEM, the only report of multiple ledges growing simultaneously is by Gamalski *et al.*^53^ on GaN nanowire growth; we suspect that behavior was due to the extremely low solubility of N in even the liquid Au–Ga catalyst which makes the situation very similar to the low solubility VSS case we discuss here for GaAs, in turn enabling multilayer growth.

## 4. Conclusion

Vapor–solid–solid growth of GaAs nanowires is compared with the vapor–liquid–solid growth by *in situ* investigation in a TEM. The phase of the catalyst particle is a function of not just the temperature but also of thermal history, so two distinct experimental strategies were designed to separately study each of these two effects. The VSS growth rate was found to be slightly slower than the VLS growth rate, but comparable, rather than substantially slower as observed in some other systems. Earlier studies on monoatomic nanowires reported VSS growth to have shorter incubation and longer layer completion compared to VLS growth. Here, unlike the monoatomic studies, we see that the incubation time for the VSS growth of Au-seeded GaAs nanowires is not necessarily very different from the VLS counterpart. Layer completion time was found to be very similar for VLS and VSS under comparable growth conditions, indicating that the diffusion of reactants through the solid catalyst particle during VSS growth is not the growth rate limiting step. Moreover, observed differences in layer completion time and to some degree the incubation time could be largely accounted for by considering changes in the catalyst geometry. We also observed that while VLS usually proceeded as single layers, in VSS growth there is a high chance of more than one layer growing simultaneously. In VSS growth there can also be two layers nucleating simultaneously. How different the growth rate would be between VLS and VSS depends on thermal history, growth conditions, how the shape of the catalyst and nanowire evolves, the material system, *etc.* Hence, with an appropriate understanding of the growth dynamics it may be possible to exploit the advantages of VSS growth for a particular technological application while retaining the more practical higher growth rates typically associated with VLS.

## 5. Methods

### 5.1. Instrumentation

A Hitachi HF-3300S environmental transmission electron microscope (ETEM) with a cold field emission gun and a CEOS B-COR-aberration-corrector was used for growing nanowires *in situ*. The growth was performed in an open cell configuration on SiN_*x*_-based MEMS chips from Norcada. The Blaze software supplied by Hitachi was used to control the sample temperature in a ‘constant resistance mode’ where the effects of gas pressure and material deposition on the temperature were compensated by a feedback mechanism. The heating chips used had windows of electron-transparent SiN_*x*_ and also holes patterned on. The nanowire growths reported here were performed while the nanowire had grown into these hole regions and thus there is no SiN_*x*_ in the background for the images shown here.

### 5.2. Precursor supply

The ETEM was connected to a gas handling system with the MOCVD gases. Trimethylgallium (TMGa) and arsine (AsH_3_) are the precursors used here. Gas flows were controlled by mass flow controllers and pressure valves, and monitored during growth with a residual gas analyzer in the exhaust gas which had been calibrated to give partial pressures at the sample. AsH_3_ was supplied directly without any dilution. H_2_ was bubbled through the TMGa bubblers maintained at low temperature (bubbler bath temperature was −20 °C for the stepwise cooling series and −10 °C for the thermal hysteresis experiment). The TMGa/H_2_ mixture was further diluted with hydrogen and a fraction of it was flowed to the ETEM.

### 5.3. *In situ* growth

GaAs nanowires were grown with Au as seed particles. TMGa and AsH_3_ were introduced at 420 °C. The TMGa flux was briefly increased to trigger nucleation. Once nucleated the TMGa flow was stopped and we searched for nanowires which were growing towards the hole in the SiN_*x*_ and appropriately aligned – *i.e.* the nanowire–catalyst interface was parallel to the electron beam direction. Due to the relatively small tilting range available with these holders, it was not always possible to align the nanowire to a zone axis. However, from the lattice spacing along the growth direction the wires were found to grow along the 〈111〉 direction (while growing zinc blende) or the equivalent 〈0001〉 direction (while growing wurtzite). Each ‘layer’ we refer to is a ‘GaAs bilayer’ *i.e.* consists of one plane of Ga atoms and one layer of As atoms [layer forming the (111) plane in the case of zinc blende and (0001) for wurtzite]. Very often, while growing at these low temperatures, the catalyst particle topples to the side of the nanowire or the nanowire kinks. The two nanowire cases reported here did not kink during the experiment.

### 5.4. Data acquisition and measurements

Blaze software developed by Hitachi is for heating the sample and also logs the temperature. The precursor fluxes that flowed to the ETEM were monitored with a residual gas analyzer (SRS RGA 300) using mass spectrometry. The precursor flows were calibrated to find the partial pressure at the sample. The TMGa partial pressure values reported here are estimated using this calibration and the mass spectrometry values measured. Further details of the experimental setup can be found in previous publications.^42,54^

The layer growth dynamics are recorded as videos made up of a series of TEM images. The growth of each layer is identified as a dynamic change in the contrast at the interface. In the TEM image we only observe a projection of the 2-dimensional plane. Sometimes in the projection it appears to grow from one side to the other. There are also instances where we see a contrast change start at somewhere in the middle (in the projected image). Occasionally there are instances where a contrast difference is seen along the majority of the interface and just increases in strength – this could be when a layer is growing front to back (or back to front). The start or end of a layer is not always evident; under such conditions we analyze the video over and over to identify it and note down the values. The layer completion times and incubation times of individual layers are found from the starting and ending times of layers. The inaccuracy in measurement of the starting and ending of individual layers leads to the error bars in the Fig. 2a*y*-axis, Fig. 2b–d*x*-axis, Fig. 4*y*-axis, Fig. 6c*y*-axis and Fig. 6d–f*x*-axis. Naturally when the error bar is smaller than the size of the symbol used it is not visible in the figure. In Fig. 2a and 6c, when a new layer starts to grow before the previous layer has completely grown the incubation time is denoted by a blue cross (*x*).

### 5.5. Average growth rate

The average growth rate is calculated as the reciprocal of the sum of the average incubation time and average layer completion time. However, when there are multiple layers growing simultaneously the layer completion time will be modified accordingly. So here for calculating the average growth rate we use only the layer completion time and incubation times of single layer growth events only (*i.e.* the previous layer was completely grown before the layer under consideration started and this layer is completely grown before the next layer nucleates). At 290 °C and 280 °C there were only two measurements of incubation time and layer completion time, making the average growth rate a very crude estimate only. At 300 °C there was no instance of single layer growth observed and so there is no value of average growth rate given for this temperature.

### 5.6. Specifics about the stepwise cooling series experiment

Temperature ramp rates for all the steps were 3 °C s^−1^. The SiN_*x*_ heating chips bulge due to thermal expansion as a function of temperature. When the temperature is changed the sample height makes the sample out of focus. The video was not acquired when the temperature was changed and a new video was started after adjusting the sample height. This effect is lower at lower temperatures – so the delay between reaching the temperature and starting the video (or analysis) & starting video is typically smaller. So, some layer growth events were not recorded causing the *x*-axis in Fig. 2a to be discontinuous. In this specific experiment the time difference between reaching the new temperature and starting the video is as follows: 420 °C (had been at this temperature for very long), 380 °C: 103 s, 360 °C: 30 s, 340 °C: 20 s, 330 °C: 16 s, 320 °C: 9 s, 310 °C: −2 s *i.e.* we had started recording 2 s before temperature was reached, but there were no layers growing in that 2 s, 300 °C: 12 s, 290 °C: 51 s and 280 °C: 2 s. The TEM images in Fig. 3 correspond to time 18 minutes 33 s in (a), 20 minutes 10 s in (b), 25 minutes 6 s in (c), and 36 minutes 52 s in (d), where time is measured as in Fig. 1b.

Au aerosol particles of nominal sizes ∼15 nm and ∼20 nm were deposited on the SiN_*x*_ chips prior to this growth experiment. During the stepwise cooling series, the precursor partial pressures near the sample were indirectly estimated to be 6 × 10^−5^ Pa of TMGa and 0.6 Pa of AsH_3_. A custom-made double-tilt holder from Hitachi High-Technology, Canada was used for the stepwise cooling series. The precursor gases were supplied in this case using stainless steel injectors opening in the microscope pole piece gap. The TEM video (bright-field) was acquired using a Gatan OneView IS camera with an exposure time of 0.159593 s for each image frame, giving 6.27 frames per s.

### 5.7. Specifics about the thermal hysteresis experiment

As mentioned previously, after nucleating the nanowires the temperature was initially decreased and then increased to compare VLS and VSS at the same temperature. Temperature ramps were done with the microscope gun valve closed during this experiment. The ramp rate was 1, 5 and 1 °C s^−1^ for the initial 280 °C (VLS), 260 °C and the final 280 °C (VSS) respectively. The time delay between reaching the new temperature and starting the recording was 100 s, 191 s and 134 s respectively. At the beginning of the first 280 °C (VLS) growth, an atomically thin ordered surface was present on the left and right sides of the catalyst–vapor interface. A couple of layers grew with this ordered surface but these layers are not included in Fig. 6. In principle, the particle would have solidified if we had maintained the system at 280 °C (VLS) for an extended period of time. However, since at these low temperatures nanowires were very prone to kinking or toppling of the catalyst particle, waiting till the particle solidifies on its own was a risky option, and hence the temperature was decreased significantly to solidify faster.

Au aerosol particles of nominal sizes ∼30 nm were deposited on the SiN_*x*_ chips prior to this growth experiment. For this experiment, a single-tilt holder with two separate microtubes running within the holder to release the precursors very close to the sample was used. The holder and the gas-handling system are connected by a polymer-coated thin quartz tube (PEEKSil) from Trajan Scientific. Precursor pressures were 1.5 × 10^−4^ Pa of TMGa and 1.1 Pa of AsH_3_. The video was recorded using an AMT XR401 sCMOS camera at a frame rate of 18 fps.

## Data availability

More data that support the findings of this study are available from the corresponding author upon request.

## Author contributions

C. B. M. conceived the project and planned the experiments. The experiments were performed by C. B. M. along with D. J. or M. T. or anyone in the research group of K. A. D. Data analysis was performed by C. B. M. All the authors discussed the results and contributed to the concepts discussed in this article.

## Conflicts of interest

There are no conflicts to declare.

## Supplementary Material

NA-003-D1NA00345C-s001

NA-003-D1NA00345C-s002

NA-003-D1NA00345C-s003
