# Supplementary material for: Vapor–solid–solid growth dynamics in GaAs nanowires
Source: Nanoscale Adv. 2021 Aug 5;3(20):5928–40. doi: 10.1039/d1na00345c (PMC9418180; doi:10.1039/d1na00345c)
Supplement: NA-003-D1NA00345C-s003 [file NA-003-D1NA00345C-s003.pdf]

Electronic Supplementary Material (ESI) for Nanoscale Advances.
This journal is © The Royal Society of Chemistry 2021

Supporting Information:
Vapor-solid-solid growth dynamics in GaAs nanowires

Carina B. Maliakkal[1,2,3], Marcus Tornberg[1,2,3], Daniel Jacobsson[1,3,4], Sebastian Lehmann[2,3], and Kimberly A. Dick[1,2,3]*

[1]Centre for Analysis and Synthesis, Lund University, Box 124, 22100, Lund, Sweden.

[2]Solid State Physics, Lund University, Box 118, 22100, Lund, Sweden.

[3]NanoLund, Lund University, 22100, Lund, Sweden.

[4]National Center for High Resolution Electron Microscopy, Lund University, Box 124, 22100, Lund, Sweden.

*E-mail: carina_babu.maliakkal@chem.lu.se

S1) Average incubation and layer completion time for the stepwise cooling series

The Figure 2 a of the main article showed incubation and layer completion time individual layer growth events. Figure S1 shown here is the averaged value at each temperature. The first few layers grown at 310 °C (i.e. temperature where catalyst solidified) are not included in this averaging (the first layer at 310 °C had grown while the catalyst was liquid and the first two layers soon after catalyst solidification showed a reduced incubation time due to the transition).

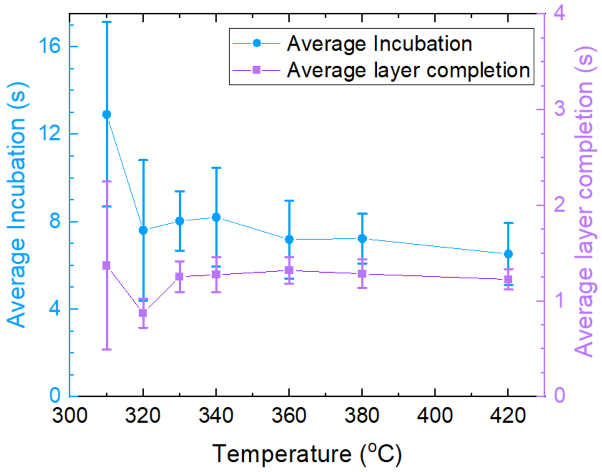

Figure S1. Average incubation and layer completion time at each growth temperature for the stepwise cooling series. Y-axis are in linear scale.

S2) Individual layer growth events at 310 ^{o}C alone in stepwise cooling series

The catalyst was initially a liquid at this temperature. The first layer at 310 ^{o}C grew while the catalyst was still a liquid. After a few seconds, the catalyst solidified and a new layer nucleated at the same time (within the 0.16 s temporal resolution of the video). The image where we observed solidification is shown in Figure S3 b. The image just before is given in Figure S3 a. (These images show the raw data, without any image rotation.) The first two layers that nucleated soon after solidification showed a reduced incubation time due to the transition. We observed two occasions of the formation of twinned zinc blende layer. The first was a single

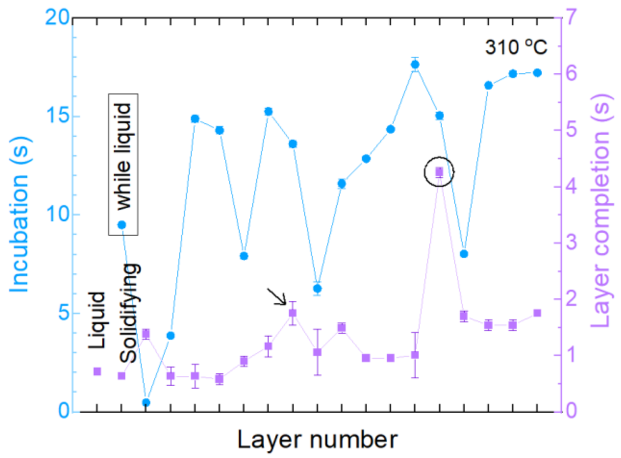

Figure S2. Layer growth at 310 ºC. Y-axis are in linear scale. The black arrow indicates a single layer growing as a twin. The black circle indicates a twinned double layer.

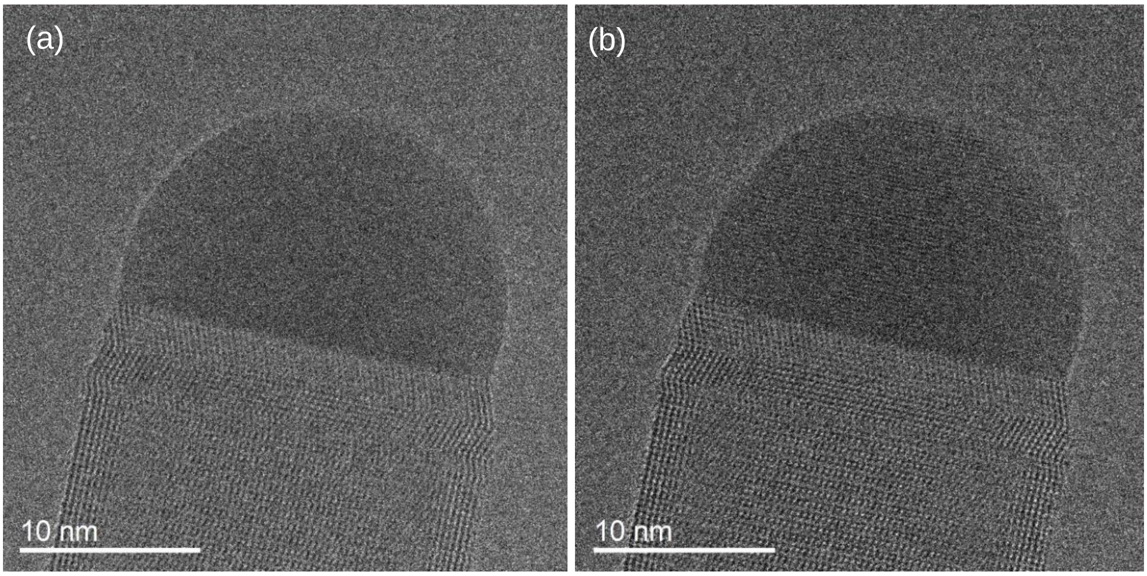

Figure S3. (a) The video frame just before solidification. The very next frame in the video is given as (b). In (b) a crystalline lattice is clearly visible. The solidification was identified by the observation of a lattice structure in the catalyst. (Exposure time of each image in the video was 0.1596s, corresponding to 6.27 fps.)

bilayer and showed a slightly higher value of layer completion time than the other layers (marked with a black arrow in Figure S2). The second instance that grew in the twinned direction was two bilayers thick (marked with black circle in Figure S2). This double bilayer was extremely slow to grow and indicates that if due to some reason multiple layers are growing together then the growth of each layer is much slower than if just one layer was growing at a time (which is not surprising).

S3) Diameter evolution of nanowire shown in stepwise cooling series

The average size of Au nanoparticles deposited on the growth chip was around 20 nm in diameter. While the nanowire was growing in the zinc blende structure the catalyst size was changing. The diameter of the catalyst-nanowire interface at 320 °C (VLS) and 310 °C (VSS), along with the incubation and layer completion times, are shown in Figure S4. The diameter is measured just before the beginning of that layer. Except at the transition stage due to solidification, at 310 °C the diameter of the solid catalyst kept increasing while the height consistently kept decreasing. The system reshaping might be driven by the formation of low-energy facets. Such drastic solid catalyst reshaping was observed in different experiments for relatively smaller Au seed (diameter ~ 20 nm). No significant reshaping was observed for 30 nm Au seeds, as can be seen in section S5.

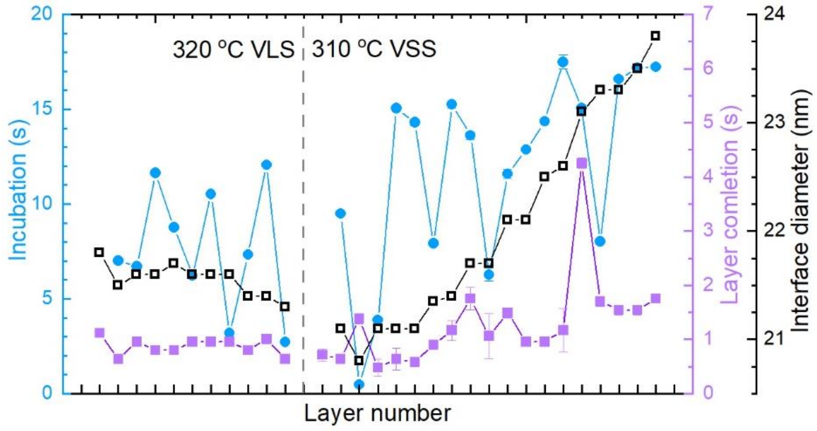

Figure S4. The diamter of the catalyst-nanowire interface at 320 °C (VLS) and 310 °C (VSS except the first layer) plotted along with the layer growth times.

As discussed in the manuscript the diameter of the catalyst-nanowire interface remained the same during the wurtzite phase growth that happened in the VLS mode before the solidification of the catalyst. Some representative images are shown below.

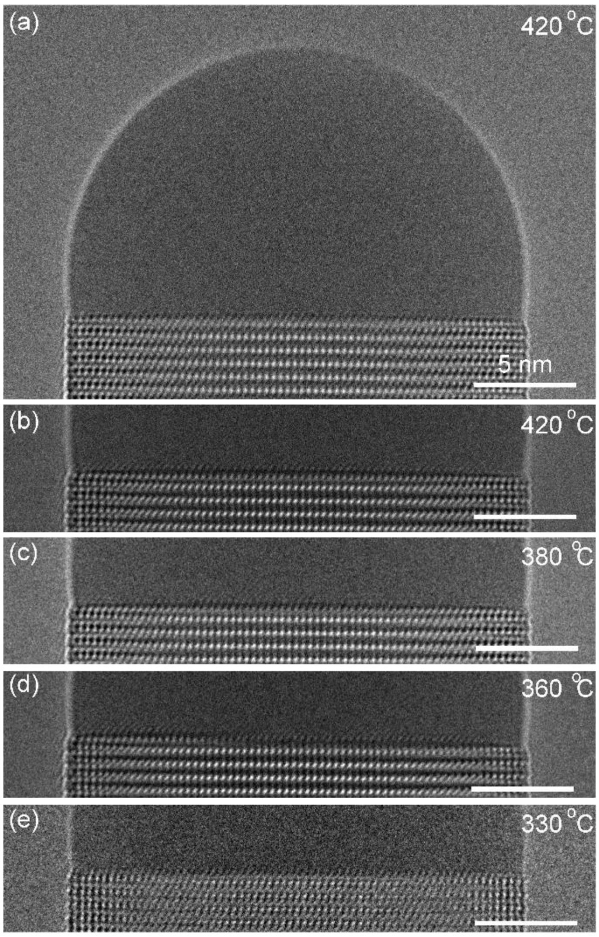

Figure S5. A few representative images in series of the catalyst-diameter interface are shown. All these images are at the same magnification, with the scale bar corresponding to 5 nm. The diamter at the interface is rather invariant during this part of growth. The time gap between (a) and € is about 11 minutes. The temperature at which these images captured in situ during growth are given on the top right corner.

S4) Geometry based model for layer completion time

We try to understand the increase of layer completion time with increasing interface diameter by a simple geometric model. There could be other factors also related to the layer completion time, however, here we look at the geometry alone. Due to the negligible surface diffusivity, arsenic atoms reach the catalyst by direct impingement on the catalyst surface.[1] Thus the number of As atoms being adsorbed is proportional to the surface area of the catalyst exposed to the surface. Arsenic desorption is also proportional to the surface area.[2] Thus, the layer completion time would be inversely proportional to the catalyst-vapor surface area. Since the GaAs layer is formed at the catalyst-nanowire interface, the layer completion time varies proportionally to the interface area. For the VSS case the catalyst shape can be very crudely approximated to a cylinder

with diameter D. (Note that the actual catalyst shape is not of a cylinder, but more complex and changing with time and would thus involve many more parameters.) We assume that while the catalyst is flattening the catalyst volume (V) remains unchanged. According to this simplistic model the layer completion would vary as $\frac{1}{1+\frac{16\ V}{\pi\ D^3}}$, like shown below. The plot shows a trend similar to that in Figure 4a of the manuscript.

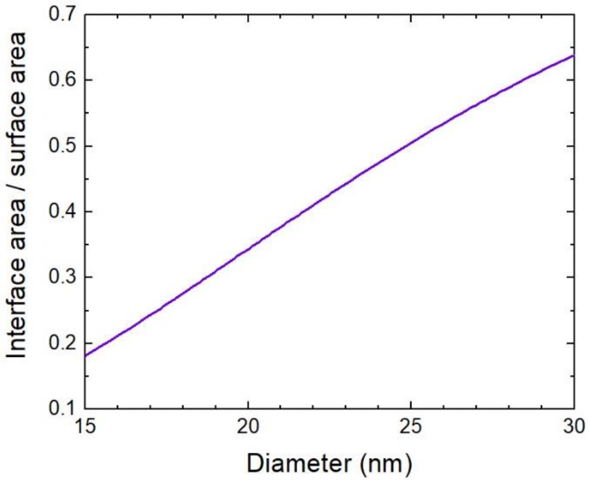

Figure S6. NW-catalyst interface area divided by the catalyst surface area exposed to the ambient vapor plotted as a function of the cylinder diameter with a fixed catalyst volume of 3000 nm^3.

S5) More images of the nanowire shown in thermal hysterisis experiment

The average size of Au nanoparticles deposited on the growth chip was around 30 nm in diameter. Some representative images of the nanowire shows no drastic flattening of the catalyst, unlike what is seen in Figure S4. Another example of a nanowire, again of similar catalyst size no obvious catalyst flattening is shown in Fig. S8.

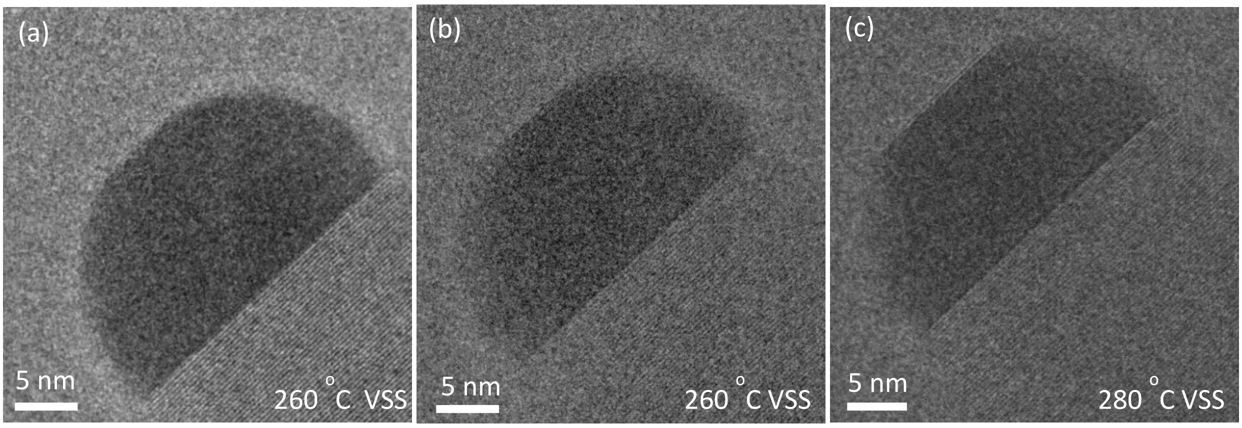

Figure S7: A series of images of the nanowire shown in Figure 6 of the manuscript. (a) is after the solidification, (b) is about 11 minutes later, (c) is even later where the temperature was also increased by 20 ^{o}C.

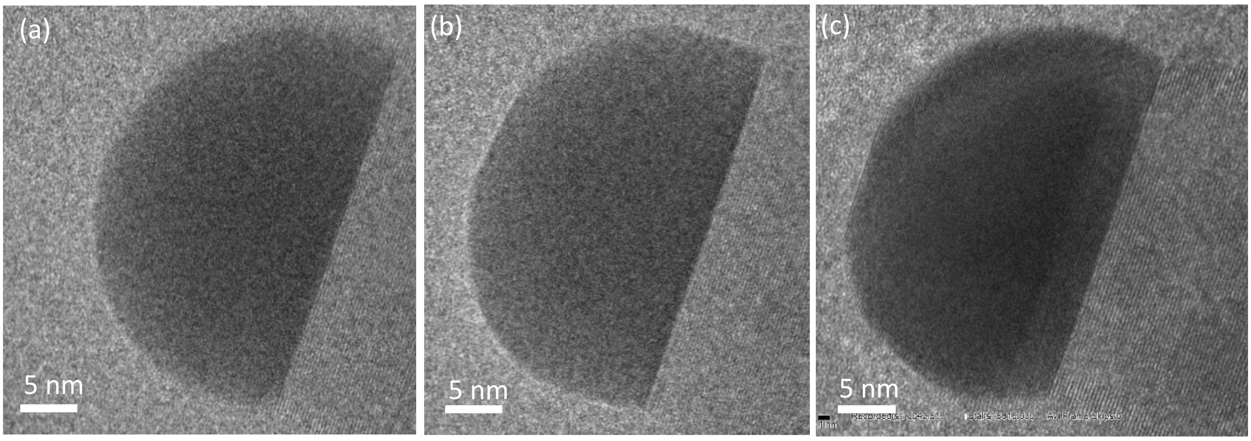

Figure S8: Another example of a solid catalyst which was obtained from Au nanoparticle deposition with 30 nm average diameter. Similar to Figure S7, this particle also did not show any drastic flattening. (a) shows the image of the nanowire growing in the VLS mode just before solidification of the catalyst particle. Images soon after solidification is shown in (b) and a much later image is shown in (c). (This experiment was performed of a SiN_x grid without any holes, and so there is SiN_x above/below the nanowire. These images were acquired at a set temperature of 280 ^oC, but with a 'constant power' mode of blaze where the power supplied to the sample for heating is not corrected by any feedback loop for compensating for material deposition. Hence the temperature value is not meaningful. Note that in all other experiments shown in the main article or the supporting information the 'constant resistance' mode was used where the temperature values are reliable.)

References

1. Ramdani, M. R., Harmand, J. C., Glas, F., Patriarche, G. & Travers, L. Arsenic Pathways in Self-Catalyzed Growth of GaAs Nanowires. *Cryst. Growth Des.* **13**, 91–96 (2013).
2. Dubrovskii, V. G. Theory of VLS Growth of Compound Semiconductors. **93**, 1–78 (2015).
